# *Ecklonia cava* Polyphenol Has a Protective Effect against Ethanol-Induced Liver Injury in a Cyclic AMP-Dependent Manner

**DOI:** 10.3390/md13063877

**Published:** 2015-06-18

**Authors:** Haruka Yamashita, Mayu Goto, Isao Matsui-Yuasa, Akiko Kojima-Yuasa

**Affiliations:** Department of Food and Human Health Sciences, Graduate School of Human Life Science, Osaka City University, 3-3-138 Sugimoto, Sumiyoshi-ku, Osaka 558-8585, Japan; E-Mails: yuzu_chiffon_o712@yahoo.co.jp (H.Y.); m14hat0Q07@st.osaka-cu.ac.jp (M.G.); yuasa-i@hotmail.co.jp (I.M.-Y.)

**Keywords:** *Ecklonia cava* polyphenol, cytochrome P450 2E1, alcohol dehydrogenase, reactive oxygen species, cyclic AMP, hepatocytes

## Abstract

Previously, we showed that *Ecklonia cava* polyphenol (ECP) treatment suppressed ethanol-induced increases in hepatocyte death by scavenging intracellular reactive oxygen species (ROS) and maintaining intracellular glutathione levels. Here, we examined the effects of ECP on the activities of alcohol-metabolizing enzymes and their regulating mechanisms in ethanol-treated hepatocytes. Isolated hepatocytes were incubated with or without 100 mM ethanol. ECP was dissolved in dimethylsulfoxide. ECP was added to cultured cells that had been incubated with or without ethanol. The cells were incubated for 0–24 h. In cultured hepatocytes, the ECP treatment with ethanol inhibited cytochrome P450 2E1 (CYP2E1) expression and activity, which is related to the production of ROS when large quantities of ethanol are oxidized. On the other hand, ECP treatment with ethanol increased the activity of alcohol dehydrogenase (ADH) and aldehyde dehydrogenase. These changes in activities of CYP2E1 and ADH were suppressed by treatment with H89, an inhibitor of protein kinase A. ECP treatment with ethanol enhanced cyclic AMP concentrations compared with those of control cells. ECP may be a candidate for preventing ethanol-induced liver injury via regulating alcohol metabolic enzymes in a cyclic AMP-dependent manner.

## 1. Introduction

Chronic alcohol abuse is a significant cause of cirrhosis and liver failure in adults worldwide. Alcoholic liver disease is a pathological process characterized by progressive liver damage that leads to steatosis, steatohepatitis, fibrosis and, finally, cirrhosis. Eventually, cirrhosis may progress to hepatic decompensation and hepatocellular cancer [1,2,3]. Oxidative stress plays an important role in this process [4,5].

*Ecklonia cava*, a brown alga produced in large quantities on Juju Island, Korea, is popular in Japan and Korea as a food, an animal feed, a fertilizer, and a drug. *Ecklonia cava* contains various phlorotannins, with a wide range of polarities and molecular weights (126 Da–650 kDa) [6]. Ahn *et al.* investigated the potential antioxidant activities of three phlorotannins (phloroglucinol, eckol and dieckol) purified from *Ecklonia cava* and found that these compounds efficiently scavenge radicals. Eckol is a superior free radical scavenger and an inhibitor of DNA damage [7]. Many researchers have reported that *Ecklonia* species exhibit significant scavenging activity [8], anti-plasmin activity [9], anti-mutagenic activity [10], bactericidal activity [11], HIV-1 reverse transcriptase and protease inhibition [12], anti-tyrosinase activity [13] and anti-fibrosis activity [14]. The total amount of polyphenolic compounds in *Ecklonia cava* is reportedly higher than in other brown seaweeds [15]. Recently, we showed that *Ecklonia cava* polyphenol (ECP) treatment suppressed ethanol-induced increases in hepatocyte death by scavenging the reactive oxygen species (ROS) and maintaining intracellular glutathione levels. This protection against ethanol-induced liver injury was confirmed using *in vivo* experiments. We demonstrated that aspartate aminotransferase (AST) and alanine aminotransferase (ALT) were released into the blood in rats treated with both ethanol and tetrachloride. Increased serum AST and ALT activities were attenuated by administrating ECP [16]. These results suggest that ECP could prevent ethanol-induced liver injury.

Alcohol is mainly metabolized in the liver in two steps. The first step is the oxidation to acetaldehyde by three enzymes: alcohol dehydrogenase (ADH) in the cytosol, cytochrome P450 2E1 (CYP2E1) in the microsome, and catalase in the peroxisome. Only the first two pathways are of practical significance. ADH is used in the degradation of limited quantities of alcohol, while alcohol-induced CYP2E1 takes place after excessive alcohol intake [17]. The second step is the oxidation of acetaldehyde to acetate. Acetaldehyde is oxidized mainly by aldehyde dehydrogenase (ALDH), which is a mitochondrial, NAD^+^-dependent enzyme [18]. Cyclic AMP (cAMP) is a second messenger in cell signaling. Potter *et al.* observed that the addition of dibutyryl cAMP to primary hepatocytes in culture increased ADH mRNA and ADH activity [19]. On the other hand, cAMP-dependent phosphorylation of CYP2E1 leads to a reduction of its activity [20]. These results suggest that cAMP is involved in the metabolism of ethanol.

In this study, we examined the effects of ECP on intracellular cAMP concentrations and the activities of alcohol metabolic enzymes in ethanol-treated hepatocytes.

## 2. Results

### 2.1. The Effects of ECP on the Cell Viability of Ethanol-Treated Hepatocytes

We previously demonstrated that treatment with 100 mM ethanol for 24 h significantly decreased cell viability compared with control cells [16]. Here, we measured the cell viability of hepatocytes treated with 100 mM ethanol, with or without various concentrations of ECP, to investigate the effects of the ECP concentration on hepatocyte cell viability. As shown in Table 1, a dose of 6.25–25 μg/mL ECP significantly prevented cell death in the hepatocytes treated with 100 mM ethanol. Therefore, the following experiments were carried out with 100 mM ethanol and with 6.25 μg/mL ECP.

**Table 1:** Effects of *Ecklonia cava* polyphenol (ECP) on ethanol-treated hepatocytes.

Groups	Cell Viability (% of Control)
Control	100.0 ± 6.7
100 mM ethanol	82.5 ± 4.8 **
100 mM ethanol + 3.1 μg/mL ECP	84.8 ± 5.3 **
100 mM ethanol + 6.25 μg/mL ECP	97.2 ± 5.3
100 mM ethanol + 12.5 μg/mL ECP	97.2 ± 8.2
100 mM ethanol + 25 μg/mL ECP	95.7 ± 5.5
100 mM ethanol + 50 μg/mL ECP	89.6 ± 4.7

Hepatocytes were incubated with 100 mM ethanol, with or without various concentrations of ECP. Cell viability was measured by the Neutral Red assay as described in the Materials and Methods section. Data are presented as the mean ± S.D. of three experiments (** *p* < 0.01 compared with control cells).

### 2.2. The Effects of ECP on ROS Levels of Ethanol-Treated Hepatocytes

**Figure 1:**
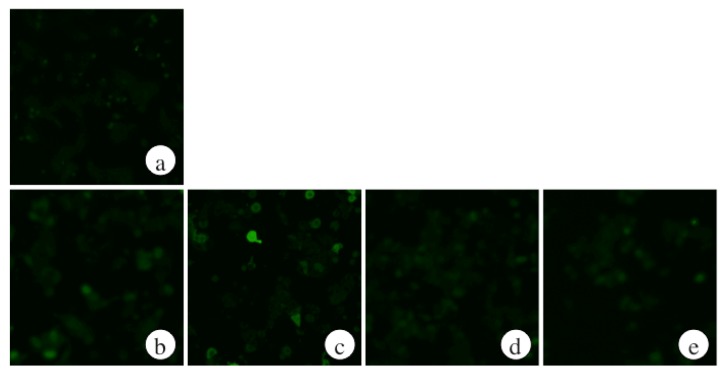

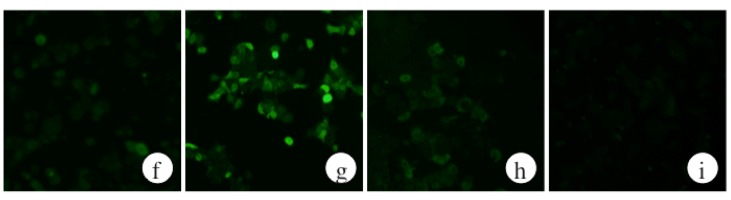
Effects of *Ecklonia cava* polyphenol (ECP) on the ethanol-induced increases in intracellular ROS formation in hepatocytes. Hepatocytes were incubated for 0–9 h with 100 mM ethanol, with or without ECP (6.25 μg/mL). ROS formation was detected as described in the Materials and Methods section. (**a**) Control, 0 h; (**b**) Control, 6 h; (**c**) 100 mM ethanol, 6 h; (**d**) 100 mM ethanol plus 6.25 μg/mL ECP, 6 h; (**e**) 6.25 μg/mL ECP, 6 h; (**f**) Control, 9 h; (**g**) 100 mM ethanol, 9 h; (**h**) 100 mM ethanol plus 6.25 μg/mL ECP, 9 h; (**i**) 6.25 μg/mL ECP, 9 h.

Ethanol-induced liver injury is characterized by the increased formation of ROS [21]. Therefore, we measured intracellular ROS levels using DCHF-DA, which is converted to highly fluorescent 2′,7′-dichlorodihydrofluorescein in the presence of intracellular ROS. Hepatocytes were incubated for 6 and 9 h with 100 mM ethanol, with or without 6.25 μg/mL ECP. Exposure to 100 mM ethanol caused an increase in ROS. However, ECP treatment maintained intracellular ROS levels below those of control cells (Figure 1).

### 2.3. The Effects of ECP on CYP2E1 Activities of Ethanol-Treated Hepatocytes

**Figure 2:**
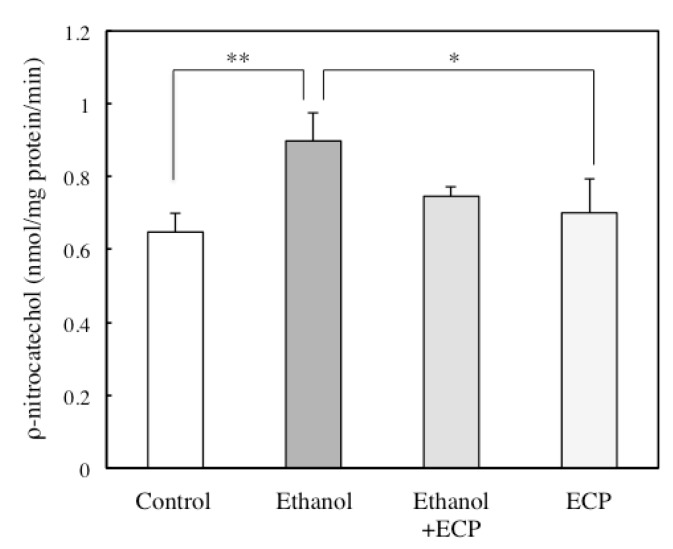
Effects of *Ecklonia cava* polyphenol (ECP) on the ethanol-induced increases in CYP2E1 expression in hepatocytes. Hepatocytes were incubated for 9 h, with or without 100 mM ethanol and ECP (6.25 μg/mL). CYP2E1 activity analysis was performed as described in the Materials and Methods section. Data are presented as the mean ± S.D. of three separate experiments (* *p* < 0.05; ** *p* < 0.01).

Induction of CYP2E1 in ethanol-treated hepatocytes is an important contributor to ethanol-induced oxidative stress [21]. We examined the effects of ECP on ethanol-induced increases in CYP2E1 activity using a PNP assay. The ethanol-treated hepatocytes significantly increased the CYP2E1 activity over the 9-h incubation period. In contrast, ECP treatment suppressed the ethanol-induced increases in CYP2E1 activity to the levels of the control cells (Figure 2).

### 2.4. The Effects of ECP on ADH and ALDH Activities of Ethanol-Treated Hepatocytes

**Figure 3:**
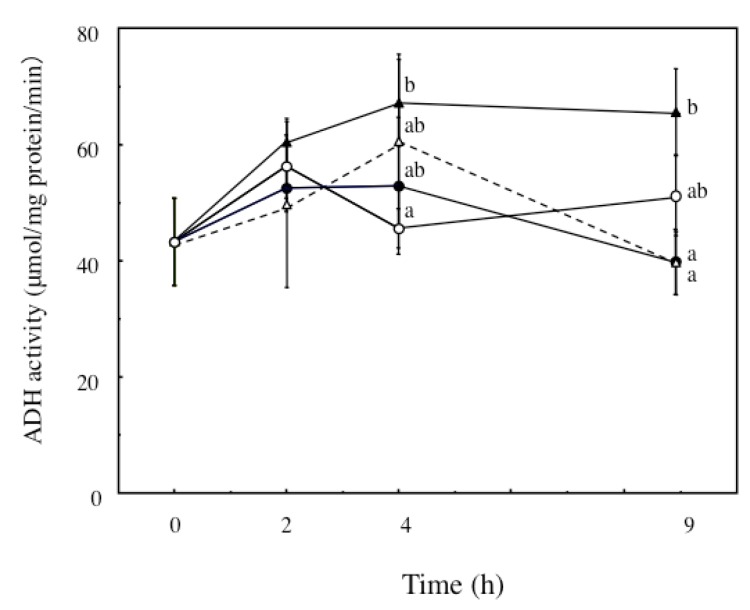
Effects of *Ecklonia cava* polyphenol (ECP) on the ethanol-induced increases in ADH activity in hepatocytes. Hepatocytes were incubated for 0–9 h with 100 mM, ethanol with or without ECP (6.25 μg/mL). ADH activity analysis was performed as described in the Materials and Methods section. Data are presented as the mean ± S.D. of three separate experiments. Values without a common letter are significantly different (*p* < 0.05 at 4 h, *p* < 0.01 at 9 h). ○: Control; ●: 100 mM ethanol; ▲: 100 mM ethanol plus 6.25 μg/mL ECP; △: 6.25 μg/mL ECP.

**Figure 4:**
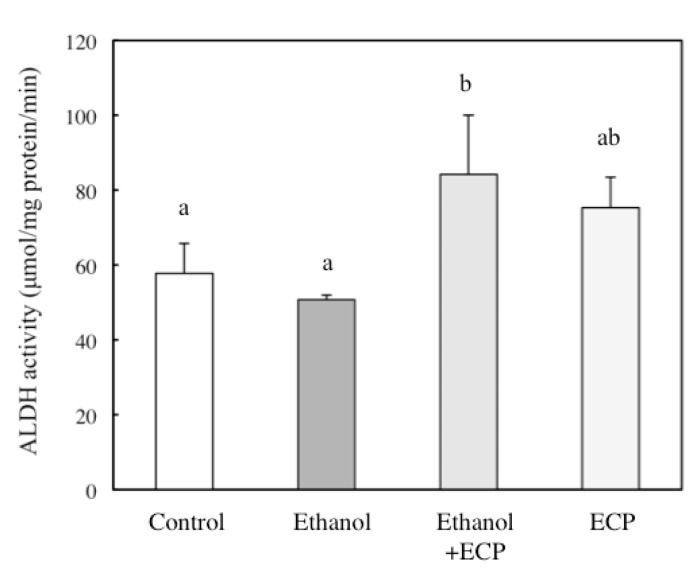
Effects of *Ecklonia cava* polyphenol (ECP) on the ethanol-induced increase in ALDH activity in hepatocytes. Hepatocytes were incubated for 4 h with 100 mM ethanol, with or without ECP (6.25 μg/mL). ALDH activity analysis was performed as described in the Materials and Methods section. Data are presented as the mean ± S.D. of three separate experiments. Values without a common letter are significantly different (*p* < 0.05).

To determine their potential protective mechanisms against ethanol-induced hepatotoxicity, we examined the activities of the major alcohol metabolizing enzymes ADH and ALDH. ECP treatment significantly enhanced ADH activity in these cells over a period of 2, 4 and 9 h (Figure 3). Furthermore, ALDH activity was also significantly enhanced by 6.25 μg/mL ECP after a 4-h incubation with ECP (Figure 4).

### 2.5. The Effects of Protein Kinase A Inhibition on CYP2E1 and ADH Activities of Ethanol and ECP-Treated Hepatocytes

**Figure 5:**
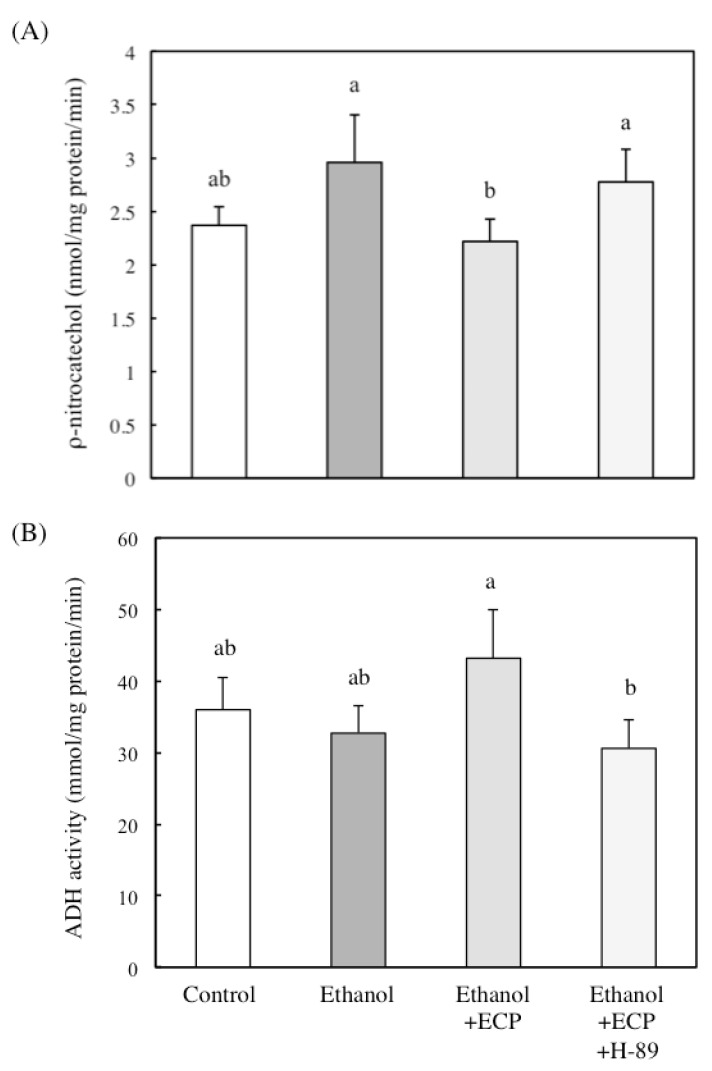
Effects of protein kinase A inhibition on (**A**) CYP2E1 and (**B**) ADH activity of ethanol and *Ecklonia cava* polyphenol (ECP)-treated hepatocytes. Hepatocytes were incubated for 9 h with or without 100 mM ethanol and ECP (6.25 μg/mL). CYP2E1 and ADH activities were assayed as described in the Materials and Methods section. Data are presented as the mean ± S.D. of three separate experiments. Values without a common letter are significantly different (*p* < 0.05).

Potter *et al.* reported that cAMP induced ADH and suggested that this effect is mediated by the binding of C/EBPβ to the C/EBP site [19]. It has been reported that CYP2E1 activity is controlled by hormones posttranslationally, resulting in its phosphorylation on Ser129 by cAMP-dependent protein kinase and degradation of the enzyme by the microsomal Mg21-ATP-dependent proteolytic system [22]. Therefore, to know whether ECP regulates the activity of ADH and CYP2E1 via cAMP/protein kinase A signaling, we examined the effects of H89, an inhibitor of protein kinase A. Treatment with H89 increased CYP2E1 activity in ethanol- and ECP-treated hepatocytes (Figure 5A). On the other hand, treatment with H89 inhibited the enhancement of ADH activity, which is induced by treatment with ethanol and ECP (Figure 5B).

### 2.6. The Effects of ECP on the cAMP Concentration of Ethanol-Treated Hepatocytes

**Figure 6:**
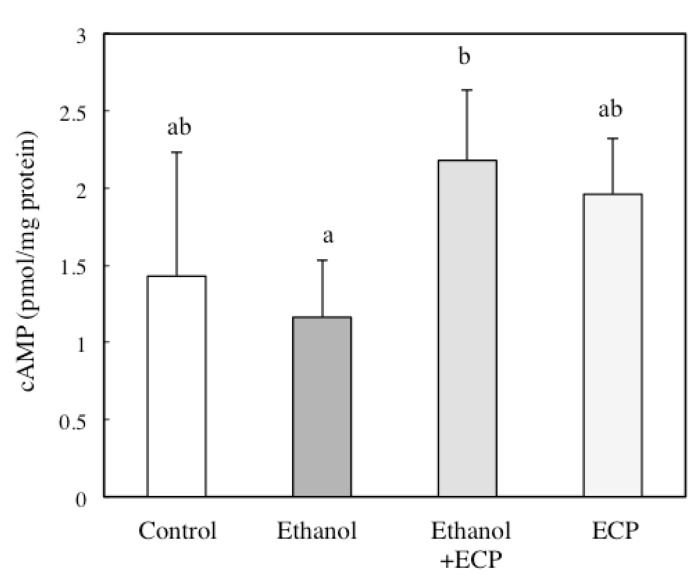
Effects of *Ecklonia cava* polyphenol (ECP) on cAMP concentration of ethanol-treated hepatocytes. Hepatocytes were incubated for 6 h, with or without 100 mM ethanol and ECP (6.25 μg/mL). cAMP concentration was assayed as described in the Materials and Methods section. Data are presented as the mean ± S.D. of three separate experiments. Values without a common letter are significantly different (*p* < 0.05).

We examined cAMP concentrations in cells incubated for 6 h with or without 100 mM ethanol and 6.25 μg/mL ECP. ECP treatment significantly increased cAMP concentrations compared with control cells (Figure 6).

**Figure 7:**
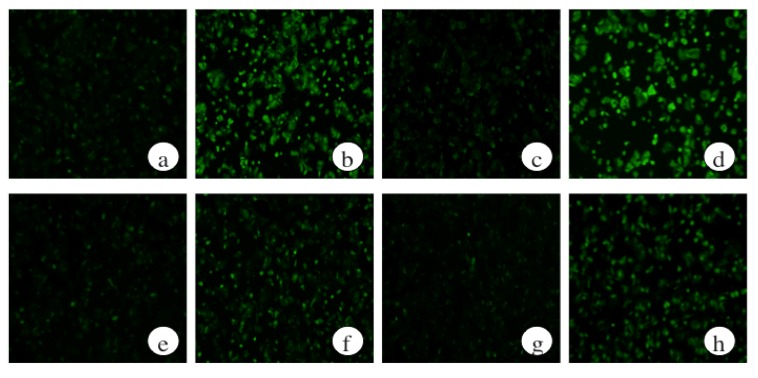
Effects of protein kinase A inhibition on intracellular ROS formation of ethanol and *Ecklonia cava* polyphenol (ECP)-treated hepatocytes. Hepatocytes were incubated for 6 or 9 h with 100 mM ethanol, with or without ECP (6.25 μg/mL). Protein kinase A inhibitor, H-89 (10 μM) was added to the medium with ethanol and ECP simultaneously. ROS formation was detected as described in the Materials and Methods section. (**a**) Control, 6 h; (**b**) 100 mM ethanol, 6 h; (**c**) 100 mM ethanol and 6.25 μg/mL ECP, 6 h; (**d**) 100 mM ethanol, 6.25 μg/mL ECP and 10 μM H-89, 6 h; (**e**) Control, 9 h; (**f**) 100 mM ethanol, 9 h; (**g**) 100 mM ethanol and 6.25 μg/mL ECP, 9 h; (**h**) 100 mM ethanol plus 6.25 μg/mL ECP plus 10 μM H-89, 9 h.

### 2.7. The Effects of a Protein Kinase A Inhibitor on the ROS Levels of Ethanol-Treated Hepatocytes

To examine whether ECP protects ethanol-induced liver injury via regulating alcohol metabolic enzymes in a cAMP-dependent manner, we tested the effects of H89 on the production of ROS in hepatocytes after ethanol and/or ECP incubation. As shown in Figure 1 and Figure 7, exposure to 100 mM ethanol and 6.25 μg/mL ECP caused a decrease in intracellular ROS levels. However, H89 treatment increased the intracellular ROS level to the levels in ethanol-alone-treated hepatocytes.

## 3. Discussion

In our previous study, we demonstrated that ECP treatment suppressed ethanol-induced increases in hepatocyte death by maintaining intracellular glutathione levels. Additionally, a suppression of ethanol-induced increases in the expression of type I collagen and α-smooth muscle actin by maintaining intracellular ROS and glutathione levels was noted. Furthermore, ECP protection against ethanol-induced liver injury was confirmed through *in vivo* experiments. We demonstrated that aspartate aminotransferase and alanine aminotransferase, which were released into the blood in ethanol plus CCl_4_-treated rats, were attenuated by administering ECP [16]. These results suggest that ECP could prevent ethanol-induced liver injury. However, the detailed mechanisms of the protective effect of ECP are not clear in ethanol-treated hepatocytes.

In the present study, we found that CYP2E1 activity was significantly increased over the 9-h incubation period in ethanol-treated hepatocytes but that ECP treatment suppressed the ethanol-induced increase in the enzyme activity to the levels of control cells. We also measured the effects of ECP on the activities of the major alcohol metabolizing enzymes, and we found that ECP treatment significantly enhanced ADH activity in the cells after 4 and 9 h of incubation and ALDH activity in the cells after 4 h of incubation. Furthermore, ethanol- and ECP-induced enhancement of ADH activity was inhibited with H-89, an inhibitor of protein kinase A (PKA). On the other hand, H-89 increased CYP2E1 activity in ethanol- and ECP-treated hepatocytes. These results suggest that the cAMP/PKA signaling pathway is involved in changes in ADH and CYP2E1 activities in ethanol- and ECP-treated hepatocytes.

The effects of acute and chronic ethanol treatment in increasing the production of ROS and enhancing the peroxidation of lipids, protein, and DNA have been demonstrated in a variety of systems, cells and species, including humans. In our previous study, we examined these ethanol-induced increases in intracellular lipid peroxidation using the TBARS assay. ECP suppressed the ethanol-induced increases in intracellular MDA levels, and it maintained the levels below those of the control cells [16]. ROS have been implicated in many major diseases. It is well known that CYP2E1 overexpression is induced by chronic alcohol intake, resulting in ROS overgeneration [23], impairment of mitochondrial function [24], and inhibition of fatty acid oxidation [25]. Lu *et al.* have reported that ethanol caused oxidative stress in wild-type mice, but that ethanol-induced oxidative stress was decreased in CYP2E1 knockout mice [26]. It has been reported that use of the CYP2E1 knockout model resulted in decreased ethanol-induced MDA accumulation and glutathione depletion [27]. These results suggested that CYP2E1 plays a critical role in increased oxidative stress. Therefore, it is widely thought that the inhibition of CYP2E1 overexpression contributes to the decrease in various impairments in hepatocytes. The present study demonstrated that ECP treatment maintained CYP2E1 activity below that of control cells (Figure 2), suggesting that ECP prevented various ethanol-induced impairments in hepatocytes by suppressing the increased activity of CYP2E1.

There are some reports that the cAMP/PKA signaling pathway regulates the activities of ADH and CYP2E1 in ethanol metabolism. Potter *et al.* demonstrated that administration of theophylline to rats, which inhibits cAMP phosphodiesterase and, hence, increases endogenous cAMP, or the addition of dibutyryl cAMP to hepatocyte culture, increased ADH activity. On the other hand, cAMP-dependent phosphorylation of CYP2E1 leads to a reduction in CYP2E1 activity [19]. Furthermore, we have shown that intracellular glutathione (GSH) levels in the control hepatocytes increased and peaked at 8 h, but no GSH peak was observed for the ethanol-treated hepatocytes at 8 h. However, an identical peak to that observed for control hepatocytes was observed for the ethanol- and ECP-treated hepatocytes [16]. Moreover, Zhu *et al.*, have shown that treatment with cyanidine-3-*O*-β-glucoside, a flavonoid, increased GSH synthesis in the liver of diabetic mice through the PKA signaling pathway [28]. Furthermore, the suppression of ROS formation by ECP in ethanol-induced hepatocytes was inhibited with H-89, an inhibitor of PKA, showing that ROS formation is regulated by the cAMP/PKA signaling pathway. These results suggest that ROS formation is regulated by the cAMP/PKA signaling pathway and that ECP may prevent ethanol-induced liver injury via regulating alcohol metabolic enzymes in a cAMP-dependent manner.

Nagy and DeSilva demonstrated that chronic treatment (48 h) with ethanol caused a biphasic effect on cAMP production in the presence of a submaximally active concentration of glucagon. Concentrations of 20–50 mM ethanol inhibited glucagon-receptor-dependent cAMP production, whereas 100–200 mM ethanol stimulated cAMP production [29]. In our experiment, hepatocytes were treated with 100 mM ethanol in the absence of glucagon for 6 h. In this condition, the production of cAMP was not enhanced with 100 mM ethanol. These results suggest that a long period of exposure to ethanol and/or the presence of glucagon in the medium are necessary for the stimulation of cAMP production in ethanol-treated hepatocytes.

The mechanisms by which ECP treatment increases intracellular cAMP concentrations are unclear. Reports on the relationships between constituents in ECP and cAMP formation are lacking. However, Ok *et al.* demonstrated that epigallocatechin-3-gallate increased cAMP via adenylate cyclase activation, and it subsequently phosphorylated vasodilator-stimulated phophoprotein-Ser^157^ through PKA activation on collagen-induced platelet aggregation [30]. The mechanisms of cAMP formation by ECP are under investigation.

## 4. Experimental Section

### 4.1. Materials

Williams’ Medium E and β-nicotinamide adenine dinucleotide hydrate were obtained from Sigma-Aldrich Co. (St. Louis, MO, USA). Fetal bovine serum (FBS) was purchased from Nichirei Biosciences, Inc. (Tokyo, Japan). Biotinylated goat anti-mouse immunoglobulin, biotinylated goat anti-rabbit immunoglobulin, and horseradish peroxidase-labeled streptavidin-biotin complex were obtained from DAKO A/S (Glostrup, Denmark). The rabbit anti-cytochrome P450 2E1 polyclonal antibody was obtained from Enzo Life Science, Inc. (Farmingdale, NY, USA). The goat anti-pan ADH polyclonal antibody was obtained from Everest Biotech, Ltd. (Oxfordshire, UK). The rabbit anti-human β-tubulin antibody and H-89, dihydrochloride was obtained from Santa Cruz Biotechnology, Inc. (Santa Cruz, CA, USA). The anti-actin mouse monoclonal antibody was obtained from Abcam plc. (Cambridge, UK). Ez West Lumi was from ATTO Co. (Tokyo, Japan). The other chemicals used in this study were special-grade commercial products purchased from WAKO Pure Chemical Co., LTD. (Osaka, Japan).

### 4.2. Preparation of ECP

A commercially available polyphenol extract from *Ecklonia cava* (Seapolynol, Livechem Inc., Jeju, Korea) was used. The total polyphenol content of the *Ecklonia cava* extract was 99.4%, as measured by the Folin-Ciocalteu reagent using phloroglucinol as a standard. The notable compounds in the *Ecklonia cava* extract that were identified by HPLC were dieckol (8.2%), 8,8′-bieckol (2.8%), 2-*O*-(2,4,6-trihydroxyphenyl)-6,6′-bieckol (2.1%), 6,6′-bieckol (1.5%), phlorofurofucoeckol-A (1.4%), eckol (0.6%), 2-phloroeckol (0.4%), 7-phloroeckol (0.4%) and phlorotannin A (0.4%) (Column: CAPCELL PAK ODS column (4.6 × 250 mm) (Waters); eluent: 30% aqueous MeOH; flow rate: 0.8 mL/min) [13].

### 4.3. Animals

Male Wistar rats purchased from Japan SLC, Inc. (Shizuoka, Japan) were housed at a constant temperature and were allowed free access to water and standard rat chow (LaboMR stock, Japan SLC, Inc., Shizuoka, Japan). Animal experiments followed our institutional criteria for the care and use of laboratory animals in research, which are in accordance with the guidelines for animal experimentation at Osaka City University.

### 4.4. Hepatocyte Preparation and Culture

Hepatocytes were isolated by collagenase perfusion following their removal from 10-week-old male Wistar rats anesthetized with sodium pentobarbital [31]. The viability of the isolated hepatocytes was greater than 90%, as determined by 0.2% trypan blue exclusion. The cells were plated on 35-mm plastic dishes at a density of 2.5 × 10^5^ cells/mL in 2 mL of Williams’ Medium E supplemented with 10% FBS. The cells were cultured in a humidified atmosphere (5% CO_2_/95% air) at 37 °C overnight. After pre-incubation, the cells were cultured in 10% FBS containing fresh Williams’ Medium E with different concentrations of ethanol, with or without ECP, for 0–24 h.

### 4.5. Cell Viability Assay

The cell viability of the hepatocytes was measured by the Neutral Red assay, as previously described [32]. Neutral Red stock solution (0.4% Neutral Red in water) was diluted 1:80 in phosphate buffered saline (PBS). Hepatocytes were incubated with the Neutral Red solution for 2 h at 37 °C to allow for the uptake of the lysosomal dye into viable cells. The Neutral Red solution was then removed, and the cultures were washed rapidly (in under 2.5 min) with a mixture of 1% formaldehyde-1% calcium chloride. A mixture of 1% acetic acid-50% ethanol was added to the cells at room temperature for 30 min to extract the Neutral Red from the hepatocytes. The optical density of each sample was then measured at 540 nm with a spectrophotometer. Cell viability was estimated as a percentage of the value obtained for untreated controls.

### 4.6. Western Blot Analysis of CYP2E1

Cells were harvested, washed twice with cold PBS, and then dissolved with lysis buffer X (10 mM HEPES (pH 7.6), 10 mM KCl, 0.1 mM EDTA, 0.5% Nonidet P40, 1 mM dithiothreitol, 0.5 mM phenylmethylsulfonyl fluoride). After two freeze-thaw cycles using liquid nitrogen, the cells were sonicated and centrifuged at 12,000× *g* for 10 min at 4 °C. The supernatant was collected as the cytosolic fraction. The precipitate was dissolved with lysis buffer Y (150 mM NaCl, 50 mM Tris (pH 7.2), 1 mM EDTA, 1% Nonidet P40, 10 μg/mL leupeptin, 10 µg/mL pepstatin A and 100 μg/mL phenylmethylsulfonyl fluoride) for 30 min. Finally, the solution was sonicated and centrifuged at 2000× *g* for 20 min at 4 °C, and the supernatant was collected as the nuclear fraction. Equal amounts of protein were loaded into each lane of a 10% SDS-PAGE gel. The separated proteins were blotted onto 0.45-μm polyvinylidene difluoride (PVDF) membranes (Amersham Pharmacia Biotech, Inc., Uppsala, Sweden). After blocking overnight with 0.1% Tween-20 and 5% non-fat dry milk in TBS, the membrane was incubated with an anti-CYP2E1 antibody (diluted 1:5000) for 1 h at room temperature. After washing, the membrane was incubated with biotinylated goat anti-rabbit immunoglobulin (diluted 1:2500) for 1 h at room temperature. The membrane was washed several times, and it was then incubated with horseradish peroxidase-coupled streptavidin (1:200) for 1 h at room temperature. After several washing steps, the color reaction was developed with Ez West Lumi. A densitometric analysis of the protein bands was performed using the software Ez-Capture MG (ATTO Corporation, Tokyo, Japan).

### 4.7. Intracellular ROS Formation

A relatively specific probe for hydrogen peroxide, 2′,7′-dichlorodihydrofluorescein diacetate (DCFH-DA), was used to analyze the formation of intracellular ROS, as previously described [33]. Briefly, cells were incubated with 2.4 mM DCFH-DA (5 μL) for the final 30 min of the treatment. Then, cells were washed twice with PBS. For visualization of the intracellular fluorescence, the cells were observed with a FSX100 Bio Imaging Navigator, which is an all-in-one fluorescence imaging system (Olympus Corporation, Tokyo, Japan).

### 4.8. CYP2E1 Activity Assay

Cells were harvested, washed twice with cold PBS, and then dissolved with 10 mM Tris HCl buffer (containing 0.25 M sucrose, pH 7.4). After two freeze-thaw cycles using liquid nitrogen, the cells were sonicated and centrifuged at 12,000× *g* for 20 min at 4 °C. The supernatant was collected as the S9 fraction. The activity of CYP2E1 was determined by the rate of hydroxylation of ρ-nitrophenol (PNP) [34] at 546 nm. The S9 fraction was added to 100 mM KH_2_PO_4_ (containing 0.2 mM PNP and 2.0 mM NADPH, pH 6.8), and it was incubated in a water bath at 37 °C for 20 min. The reaction was stopped using 0.6 M perchloric acid (250 μL), and 10 M NaOH (75 μL) was added to the remaining supernatant. The results were expressed as formed ρ-nitrophenol nmo/mg protein/min, and the concentration of 4-nitrocatechol was determined (ε = 10.28 mM^−1^ cm^−1^).

### 4.9. Assay of ADH and ALDH Activities

After incubation, the cells were washed twice and then dissolved with cold PBS. The debris was obtained by centrifugation at 2600× *g* for 1 min at 4 °C, and then, buffer (50 mM HEPES pH 7.5, 0.25 M sucrose, 1 mM EDTA, 1 mM dithiothreitol (DTT), 3 mM MgCl_2_, 1 mM phenylmethylsulphonyl fluoride) was added. After two freeze-thaw cycles using liquid nitrogen, the cells were sonicated and centrifuged at 12,000× *g* for 20 min at 4 °C. Finally, the supernatant was collected. ADH activity was determined at 25 °C in a 1.5 mL volume (50 mM HEPES pH 8.0, 10 mM MgCl_2_, 1 mM DTT, 300 μM NAD^+^) in the presence or absence of ethanol (50 μL). ALDH activity was assayed at 25 °C in a 1.5 mL volume (50 mM HEPES pH 8.0, 10 mM MgCl_2_, 1 mM DTT, 300 μM NAD^+^) in the presence or absence of acetaldehyde (50 μL). The reaction was started by adding ethanol or acetaldehyde, and the absorbance at 340 nm was followed in a spectrophotometer. The linear initial increase in absorbance was used to determine specific enzyme activities with an absorption coefficient of 6.2 mM cm^−1^.

### 4.10. Assay of Intracellular cAMP Levels

The cAMP levels were measured using a Cyclic AMP Direct EIA Kit (Arbor Assays, Ann Arbor, MI, USA). PC12 cells were incubated with ACA. After incubation with ACA for 30 min, the PC12 cells were collected and washed with PBS. Next, the cells were lysed in sample diluent for 10 min at room temperature. The lysed cells were sonicated on ice in a DU-250 Bioruptor, with a maximum output power of 250 W. After centrifugation at 13,000× *g* for 10 min at 4 °C, the supernatants were used for the assay. Endogenous phosphodiesterases are inactivated in the sample diluent. A clear microtiter plate, coated with an antibody to capture sheep IgG, was provided, and a neutralizing plate primer solution was added to the wells. Then, a cAMP-peroxidase conjugate was added to the wells. The binding reaction was initiated by the addition of a sheep antibody to cAMP to each well. After a 2-h incubation, the plate was washed, and the substrate was added. After a short incubation, the reaction was stopped and the intensity of the generated color was detected via a microplate reader (wavelength = 450 nm). The total protein content was measured using the Bradford method with bovine serum albumin as the standard [35].

### 4.11. Statistical Analysis

The results are presented as the mean ± SD. Statistical comparisons were performed between groups by one-way analysis of variance, and post hoc multiple comparisons were conducted using Tukey’s test. A *p*-value of less than 0.05 was considered significant.

## 5. Conclusions

In conclusion, our experiments in cultured hepatocytes demonstrated that ECP treatment with ethanol inhibited CYP2E1 expression and activity. At the same time, ECP treatment with ethanol increased the activity of ADH and ALDH. These changes in the activities of CYP2E1 and ADH were suppressed by treatment with H89, an inhibitor of PKA. ECP treatment with ethanol enhanced the cAMP concentration compared with those of control cells. These results suggested that ECP is a candidate for preventing ethanol-induced liver injury via regulation of alcohol metabolic enzymes.
